# Knowledge and skills retention following Emergency Triage, Assessment and Treatment plus Admission course for final year medical students in Rwanda: a longitudinal cohort study

**DOI:** 10.1136/archdischild-2014-306078

**Published:** 2014-06-12

**Authors:** Lisine Tuyisenge, Patrick Kyamanya, Samuel Van Steirteghem, Martin Becker, Mike English, Tom Lissauer

**Affiliations:** 1 Department of Paediatrics, University Teaching Hospital of Kigali (CHUK), Kigali, Rwanda; 2 School of Medicine, University of Rwanda, Kigali, Rwanda; 3 Department of Paediatrics, Ambroise Pare University Hospital, Mons, Belgium; 4 Department of Paediatrics, Hinchingbrooke Hospital, Hinchingbrooke, UK; 5 KEMRI-Wellcome Trust Research Programme, Nairobi, Kenya; 6 Nuffield Department of Medicine, University of Oxford, UK; 7 Department of Paediatrics, Imperial College Healthcare NHS Trust, London, UK

**Keywords:** Medical Education, Accident & Emergency, Resuscitation, Low income populations, Rwanda

## Abstract

**Aim:**

To determine whether, after the Emergency Triage, Assessment and Treatment plus Admission (ETAT+) course, a comprehensive paediatric life support course, final year medical undergraduates in Rwanda would achieve a high level of knowledge and practical skills and if these were retained. To guide further course development, student feedback was obtained.

**Methods:**

Longitudinal cohort study of knowledge and skills of all final year medical undergraduates at the University of Rwanda in academic year 2011–2012 who attended a 5-day ETAT+ course. Students completed a precourse knowledge test. Knowledge and clinical skills assessments, using standardised marking, were performed immediately postcourse and 3–9 months later. Feedback was obtained using printed questionnaires.

**Results:**

84 students attended the course and re-evaluation. Knowledge test showed a significant improvement, from median 47% to 71% correct answers (p<0.001). For two clinical skills scenarios, 98% passed both scenarios, 37% after a retake, 2% failed both scenarios. Three to nine months later, students were re-evaluated, median score for knowledge test 67%, not significantly different from postcourse (p>0.1). For clinical skills, 74% passed, with 32% requiring a retake, 8% failed after retake, 18% failed both scenarios, a significant deterioration (p<0.0001).

**Conclusions:**

Students performed well on knowledge and skills immediately after a comprehensive ETAT+ course. Knowledge was maintained 3–9 months later. Clinical skills, which require detailed sequential steps, declined, but most were able to perform them satisfactorily after feedback. The course was highly valued, but several short courses and more practical teaching were advocated.

**What is already known on this topic?**

- Improved knowledge and skills can be demonstrated by immediate postcourse evaluation after short training courses.
- Whereas knowledge is maintained, practical skills, such as resuscitation, rapidly decline within weeks or months after training.

**What this study adds?**

- Medical students in a low-income country performed well after the comprehensive 5-day ETAT+ course in knowledge and clinical skills.
- On reassessment 3–9 months later, knowledge was retained; although clinical skills deteriorated, most were able to perform them satisfactorily after minimal feedback.
- Course was highly valued and clinically relevant, but two or more shorter courses may avoid factual overload and enhance skills retention but needs evaluation.

## Introduction

Paediatric life support courses are increasingly advocated for use in low-resource and medium-resource countries to assist achieving Millennium Development Goal 4 (MDG 4), reduction in mortality rate of children less than 5 years by two-thirds between 1990 and 2015.^1^

The (Emergency Triage, Assessment and Treatment plus Admission Care (ETAT+) is a paediatric life support course developed in Kenya for hospital health professionals caring for acutely ill children.^2^ It is an intensive 5-day course covering the recognition and initial management of the 10 commonest medical causes of paediatric hospital admission in East Africa (table 1) with lectures and case scenarios.^3^ The course was introduced in 2009 into Rwanda for medical students and hospital health professionals following a request by the Ministry of Health. Rwanda anticipates achieving its MDG4 target of 54/1000 livebirths from its baseline value in 1990 of 163/1000, despite a rise to 250/1000 following the 1994 genocide.^4^ This has been achieved in spite of ranking 167/187 on the Human Development Index.^5^ However, although Rwanda has a population of almost 11 million and 449 000 births/year,^6^ it has only about 20 paediatricians, concentrated in the two University hospitals and three other major hospitals. Developing a better-trained health workforce, particularly in district hospitals, is therefore a priority. In these facilities, general doctors take responsibility for the assessment and care of sick neonates and children, usually without any specialist or senior supervision.

**Table 1:** Topics covered in ETAT+ course

Triage
Recognition of the sick child
Neonatal resuscitation
Cardiopulmonary resuscitation of children
Diarrhoea/dehydration and shock
Pneumonia
Asthma
Malaria
Malnutrition
Sepsis/meningitis
Convulsions
Hypoglycaemia
Neonatal—preterm, sepsis, jaundice, nutrition
Procedures—oxygen, intraosseous, lumbar puncture
Prescribing

ETAT+, Emergency Triage, Assessment and Treatment plus Admission.

Training young doctors already in clinical service, however, presents a major challenge. Once the doctors are working in district hospitals, it is difficult or sometimes impossible to assemble a group of them to attend a 5-day course because they are dispersed geographically and have heavy workloads and onerous on-call rotas. An efficient alternative is to provide preservice training to final year medical students during their paediatric clinical rotation. Here the concern is that the full 5-day ETAT+ course would be too advanced for their limited clinical experience and that they would have difficulty in reaching the required standard set for qualified health professionals. An evaluation of student training was therefore planned to gauge the ability of students to acquire and retain knowledge and skills. The opportunity was also taken to gain feedback to identify how best to further adapt the course to this target group. These findings may be relevant for other countries in Africa considering the introduction of the ETAT+ or other life support courses.

## Methods

Medical training in Rwanda comprises a 6-year undergraduate training programme conducted by the University of Rwanda. Previously there was no systematic training in the recognition of the sick child or paediatric life support. We therefore introduced the full 5-day ETAT+ training for all final year (year 6) medical students during their paediatric attachment between November 2011 and May 2012. The course comprised lectures (11 h), demonstration and practice of practical procedures and case scenarios (22 h), audit and quality improvement including review of selected patient case notes (3 h) and assessment and feedback and presentation of certificates (4 h).

At the start of the course, candidates completed a knowledge test comprising 60 multiple choice (MCQ) questions (50 true/false and 10 best of 5 questions). Their mark was the percentage of correct answers. At the end of the course, all candidates retook the same knowledge test and were assessed on two clinical skills scenarios that had been taught during the course, although candidates had no prior indication of this selection. Assessors had received detailed training and the senior instructor monitored assessor performance. Precourse knowledge tests were completed at home or at the start of the course with access to course material, whereas exam conditions were applied for other knowledge assessments.

The clinical skills scenarios were neonatal resuscitation and management of an acutely ill child with shock from dehydration, marked according to standardised criteria. To pass the course, candidates had to pass both scenarios; if all standardised criteria were not met in one scenario, candidates were given feedback and allowed to retake the clinical scenario; if they did not reach the required standard after the retake, the student was judged to have failed. If they failed both scenarios at their first attempt, they were judged to have failed and were not allowed to retake the scenarios.

A repeat assessment was performed soon after completion of final examinations, 3–9 months later. It comprised a knowledge test of similar format and standard, and the same two clinical scenarios, assessed using the same criteria. Attendance was voluntary; it was explained that their individual results would be collated for a study but remain confidential and not influence their final year assessment. The benefit of attending was that it would refresh their knowledge and skills, with personal feedback of their performance. They were also given a memory stick with all the ETAT+ materials including the lectures, guideline book and publications.

The scores of individual students’ precourse and postcourse and at follow-up written tests were compared using the Wilcoxon matched-pairs signed rank test, a non-parametric test as test results were not normally distributed.

In a similar way, individual students’ scenario scores postcourse and after follow-up were compared.

To compare changes with time, we allocated a score to each possible clinical skill outcome. Passing both scenarios immediately gained a score of 3; one pass with one fail followed by a pass on retest scored 2; one pass with one fail followed by a fail on retest a score of 1; and failing both scenarios initially a score of 0. The time interval in months and individual difference in scores were compared by Spearman's rank correlation.

We elicited feedback from a subsample of students using semistructured, self-administered, written questionnaires. Feedback on the courses themselves was requested on three of four occasions.

Ethics approval was not sought as the course was adopted as part of the medical training curriculum, re-attendance was voluntary and results were anonymous.

## Results

**Figure 1:**
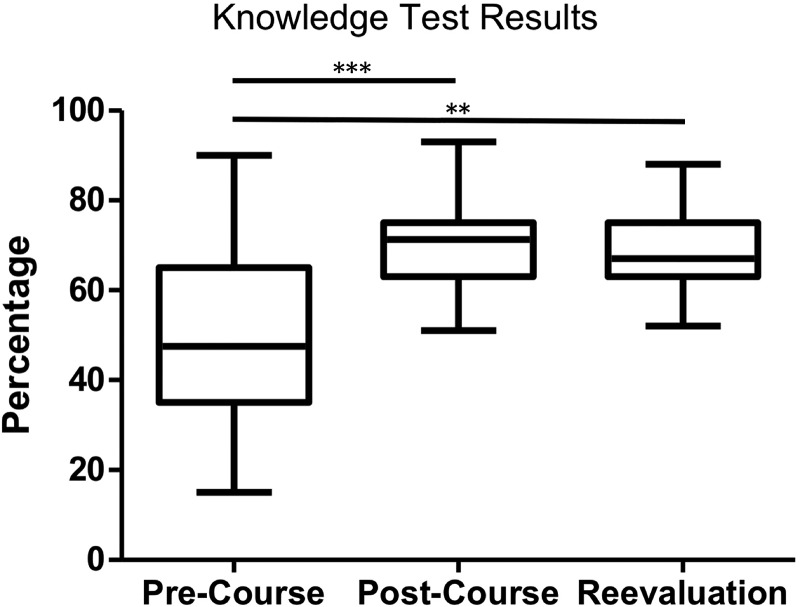
Knowledge test results showing the percentage of correct answers. Lines show median percentage, boxes demonstrate IQR and whiskers show maximum and minimum results. ***, p<0.0001, **, p>0.1 by Wilcoxon matched-pairs signed rank test.

Between November 2011 and May 2012, 88 of the 92 final year undergraduate medical students completed one of four courses; four were unable to attend the whole course and were excluded. In August 2012, 84 students (95%) were re-evaluated, 3–9 months after their course. The results of the knowledge test are shown in figure 1.

The median scores of knowledge assessments at the start and end of the course were 47% (IQR 35 to 65) and 71% (IQR 63 to 75), respectively (p<0.0001). On retesting 3–9 months later, MCQ results were similar to postcourse at 67%, (IQR 52 to 75, p>0.1).

The clinical skills assessments used were the same postcourse and at re-evaluation, although the students were not aware of this in advance. Overall, immediately after the course 98% (82/84) passed, with 61% (51/84) passing both stations on the first attempt; 37% (31/84) passing after a retake, 0% (0/84) failed on retake and 2% (2/84) failed both stations on initial testing. On re-evaluation 74% (63/84) passed, with 42% (35/84) passing both stations on the first attempt; 32% (27/84) passing after a retake, 8% (7/84) failed on retake and 18% (15/84) failed both stations on initial testing; a statistically significant deterioration in performance (p<0.0001).

**Figure 2:**
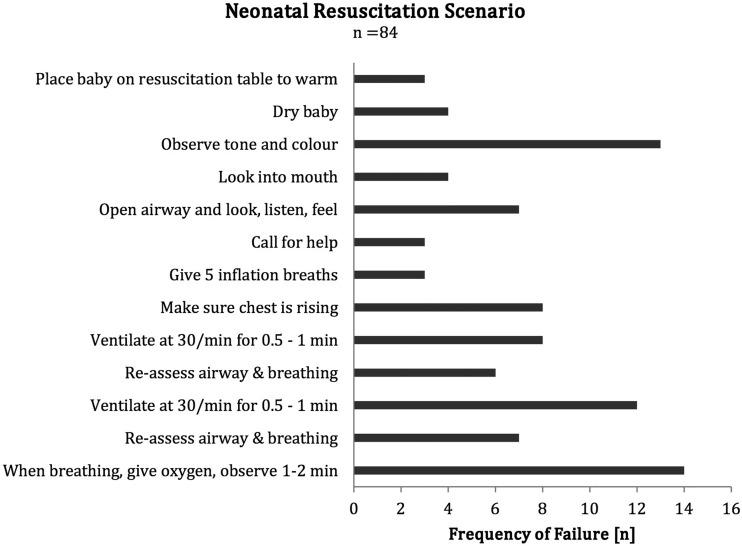
Reasons for failure of neonatal resuscitation.

**Figure 3:**
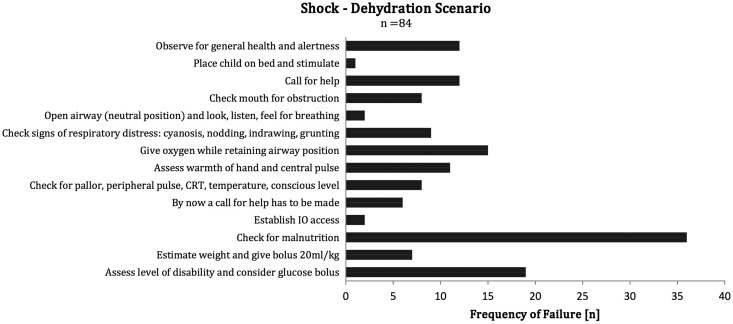
Reasons for failure of management of shock due to dehydration. CRT, capillary refill time; IO, intraosseous.

Overall, students found the newborn scenario easier in that 67% passed the assessment first time in comparison to 57% for the shock from dehydration scenario. Figures 2 and 3 show which of the 14 management steps in each scenario were omitted or done inadequately by students who failed the scenario at re-evaluation.

The relationship between the time interval and performance postcourse and re-evaluation is shown in table 2. Comparing time interval in months and difference in scores for each candidate showed no significant correlation for the knowledge test (p=0.83) or clinical skills (p=0.145) by Spearman's rank correlation.

**Table 2:** Relationship between time interval and performance postcourse and at re-evaluation

Time of course	Time interval (months)	Knowledge assessment (% correct)		Clinical scenarios							
		Postcourse (n=84)	Aug re-evaluation (n=84)	Postcourse (no. candidates=84)				August re-evaluation (no. candidates=84)			
				Pass	Pass with retake	Fail after retake	Fail	Pass	Pass with retake	Fail after retake	Fail
November	9	68	67	9	10	0	0	5	9	1	4
January	7	67	67	15	6	0	2	8	8	1	6
March	5	72	67	14	7	0	0	10	6	2	3
May	3	68	67	13	8	0	0	12	4	3	2

Four participants did not attend both assessment sessions and were excluded from these analyses. Their postcourse knowledge test was a mean of 73% and only one failed the practical skills, suggesting that non-attenders were not a cohort of poor performers.

### Feedback

Feedback postcourse (55/67, 82% returned questionnaires) showed the clinical scenarios were the most highly rated aspect of the course (80% highly satisfied, 18% satisfied), but lectures were also highly rated (59% highly satisfied, 37% satisfied). Less appreciated was a session on quality improvement involving an audit of the hospital paediatric department. Suggestions for improvement were mainly to have a longer course in two or more sessions to allow more time for clinical scenarios and practical skills, to avoid information overload and (paradoxically) to add a session on HIV infection. Many commented on the excellence of the instructors.

## Discussion

Although the potential value of paediatric life support courses in reducing morbidity and mortality of sick children is increasingly recognised, they need to be contextually appropriate rather than being transferred directly from western countries.^1^ The ETAT+ course was chosen as it has been designed for hospital health professionals in East Africa and covers the main causes of hospital admission with management recommendations tailored to local resources.^7^ It is an extension of the WHO ETAT course^8^ and is based on the principles of the WHO Integrated Management of Childhood Illness.^9^

Although primarily designed for health professionals caring for sick children in hospital, we showed that the final year medical students coped well with the course content, with a marked improvement in knowledge and a very high pass rate for clinical skills. This was in spite of their limited experience in clinical paediatrics, the intensive pace of the course and that case scenario teaching with simulation using manikins was new to them.

The students rated the content and training approach highly, especially the scenario-based teaching. They praised the organisation of the course itself and the fact that the instructors were well trained and could answer their questions directly. This is noteworthy; training in low-income countries often relies on a ‘cascade’ strategy to reach large numbers, with people trained on one course becoming trainers after a brief introduction to the teaching materials, often with no further supervision or mentorship. Yet the credibility of the educator as an opinion leader, based on their knowledge, mastery and professionalism, may be important in promoting adoption of the practices included in training^10^ and so identifying and mentoring proficient instructors is an explicit part of the ETAT+ approach.

The sessions on audit of hospital facilities, case notes and quality improvement, were not rated so highly by students who felt time could be better spent on clinical scenarios and practical skills. This is probably because they are still at an early stage of learning, focusing on clinical practice and practical procedures. Inclusion of these sessions reflect the philosophy of the ETAT+ course that knowledge and skills are not enough, they should be used to improve hospital practice and drive quality improvement.^11^ For students, these activities might be better included when preparing them to manage service provision as doctors in district hospitals.

The finding on re-evaluation after 3–9 months that the students retained their knowledge^12^ but their clinical skills declined is consistent with other studies of neonatal resuscitation^13–15^ and other life support procedures.^16–19^ The high level of knowledge retention after periods up to 9 months may, however, have been assisted by the students recently having sat their final year examinations. The commonest reasons for failing the case scenario assessment included aspects such as omitting to check for signs of severe malnutrition in a dehydrated child or not stating that they observed tone and colour during neonatal resuscitation. Such tasks are likely to be considered when observing a real child, whose state of nutrition and tone and colour would be apparent clinically, but were omitted for a low fidelity manikin. Similarly, failure to meet the exact sequence specified to pass may not necessarily result in failure of resuscitation. Therefore, despite the skills decay, we were reassured that most students could pass the practical skills assessment following brief feedback (something required by even senior clinicians in practice^20^) and are likely to have retained sufficient knowledge and skill to intervene effectively in an emergency. This does however point to the value of refresher training,^21^ something students themselves suggested in feedback.

While this evaluation confirmed the applicability and value of the 5-day ETAT+ training approach, student feedback suggested potential changes and improvements to consider and evaluate. Shortening the course, for example, to 3 days (as is done in Kenya), might make obtaining instructors and provision within the student timetable easier while reducing cost. This approach does not, however, provide for assessment and certification. Reducing the number of lectures by using precourse knowledge development strategies is one approach adopted by many courses offered in western countries, for example, the Advanced Paediatric Life Support.^22^ However, when questioned, fewer than half Rwandan medical students supported this suggestion, although we ascertained that most medical students in Rwanda owned a laptop and could access computers at the university, but this may be less available in other settings.

### Scaling up courses

Considerable sums of donor money are spent on training health workers in essential skills in all disciplines, yet preservice training is often ignored. In this first formal evaluation, we have demonstrated the feasibility and effectiveness of ETAT+ preservice training for medical students. Scaling up ETAT+ courses to other medical schools and other low-income and middle-income countries is a means to rapidly and efficiently provide knowledge and skills in a method based on adult learning theory. Local ownership and contextualisation of training is also important, the establishment of ETAT+ in Rwanda was overseen by the Rwanda Paediatric Association and the Ministry of Health and they have participated in critical reviews of the evidence supporting its content.^23^ ^24^ The programme has been strengthened and gained regional identity through an ETAT+ partnership programme involving Rwanda, Kenya and Uganda supported by the Royal College of Paediatrics and Child Health in the UK. This also supports standard setting in clinical care and improved quality of instruction.

Even good courses, however, are no substitute for health systems that enable continuous learning and improvement.^25^ Scaling up training should be considered as part of a broader strategy that ensures ready availability of guidelines linked to reminders (such as posters or on mobile electronic devices), continuing education (eg, by ad hoc mock simulations) and ongoing supervision and mentorship that may all support changes in real practices.^26^

## Conclusion

We have shown that after a 5-day ETAT+ course, final year medical students in Rwanda markedly improved their knowledge and could demonstrate life-saving practical skills during case simulations. After 3–9 months, knowledge was retained, practical skills performance declined but could usually be enhanced to reach an appropriate standard by rapid feedback. Students liked the ETAT+ course content and its delivery. Opportunities exist to further tailor approaches to delivering knowledge and skills content to optimise learning and efficiency as part of preservice training. Such training should, however, be part of a broader, systematic approach to implementing effective practices that includes regular reinforcement of clinical and life support skills.
